# Opportunities and challenges for the sustainable production of structurally complex diterpenoids in recombinant microbial systems

**DOI:** 10.3762/bjoc.13.85

**Published:** 2017-05-08

**Authors:** Katarina Kemper, Max Hirte, Markus Reinbold, Monika Fuchs, Thomas Brück

**Affiliations:** 1 Professorship for Industrial Biocatalysis, Department of Chemistry, Technical University of Munich, Lichtenbergstraße 4, 85748 Garching, Germany

**Keywords:** enzyme engineering, heterologous production in *E. coli*, metabolic pathway optimization, modular biosynthesis, plant diterpenes

## Abstract

With over 50.000 identified compounds terpenes are the largest and most structurally diverse group of natural products. They are ubiquitous in bacteria, plants, animals and fungi, conducting several biological functions such as cell wall components or defense mechanisms. Industrial applications entail among others pharmaceuticals, food additives, vitamins, fragrances, fuels and fuel additives. Central building blocks of all terpenes are the isoprenoid compounds isopentenyl diphosphate and dimethylallyl diphosphate. Bacteria like *Escherichia coli* harbor a native metabolic pathway for these isoprenoids that is quite amenable for genetic engineering. Together with recombinant terpene biosynthesis modules, they are very suitable hosts for heterologous production of high value terpenes. Yet, in contrast to the number of extracted and characterized terpenes, little is known about the specific biosynthetic enzymes that are involved especially in the formation of highly functionalized compounds. Novel approaches discussed in this review include metabolic engineering as well as site-directed mutagenesis to expand the natural terpene landscape. Focusing mainly on the validation of successful integration of engineered biosynthetic pathways into optimized terpene producing *Escherichia coli*, this review shall give an insight in recent progresses regarding manipulation of mostly diterpene synthases.

## Introduction

Isoprenoid natural products are one of the most structurally diverse groups of primary and secondary metabolites in all kinds of organisms. Moreover, they represent an invaluable source of bioactive natural products. Prominent representatives of these compounds are taxol [1] (paclitaxel, anticancer drug), artemisinin [2] (antimalarial agent) and α-pinene [3] (antibiotic, anti-inflammatory). Apart from bioactive compounds with applications as drugs/pharmaceuticals [4] or in the nutrition or agricultural sector, isoprenoids of minor structural complexity are used as bulk chemicals or fuel additives [5–6]. To identify new isoprenoids of industrial relevance essential oil extracts are screened for bioactive properties that may furnish future drugs [7–8]. Harnessing isoprenoid compounds for large-scale industrial purposes can be hampered due to low natural occurrence. Being mostly secondary metabolites isoprenoid titers in plants may be low in dependence to seasonal [9] or circadian expression [10]. On the other hand for some members simply the number of available plants may be limited like the pacific yew *Taxus brevifolia* from which paclitaxel was first extracted [11]. The chemical synthesis can be an alternative for the delivery of simpler isoprenoid structures such as carotenoids [12]. Industrially relevant total synthesis of highly oxygenated terpenes comprising several chiral centers is often more complex since usually various different reaction steps have to be performed that regularly involve cost- and workup-intensive metal-organic catalysts [13–16]. The work of McKerrall et al. on ingenol [17–18] sets an example for the difficulty of stereoselective synthesis of complex diterpenes. Additionally, semi-synthetic approaches are tainted with the equivalent issues of economic efficiency and sustainability akin to total chemical synthesis, which is often associated with toxic metal-organic chemistry, low product yields and/or insufficient purity [19–20].

A promising route for sufficient supply of industrially relevant products or their precursors is the heterologous production of plant diterpenes in well-established recombinant hosts, such as *Escherichia coli* [21–23]. Recent developments in this field will be reviewed in this work.

## Review

### Biosynthesis of diterpenes and transfer to heterologous production system

Integration of biosynthetic gene clusters from plants into a bacterial host is often not trivial due to complex metabolic coherences. The essential steps in establishing successful production of diterpenoid carbohydrate backbones in heterologous systems can be partitioned into three following areas:

1: formation of central isoprenoid precursors,

2: combination of C5-building blocks to linear isoprenyl diphosphates and

3: cyclization or condensation reaction by synthase enzyme(s).

General catalytic processes involved in these steps will be presented briefly in the following section. Selected elements of the distinct pathways will be discussed in more detail when describing the metabolic engineering of a bacterial host.

### Precursor formation

**Scheme 1:**
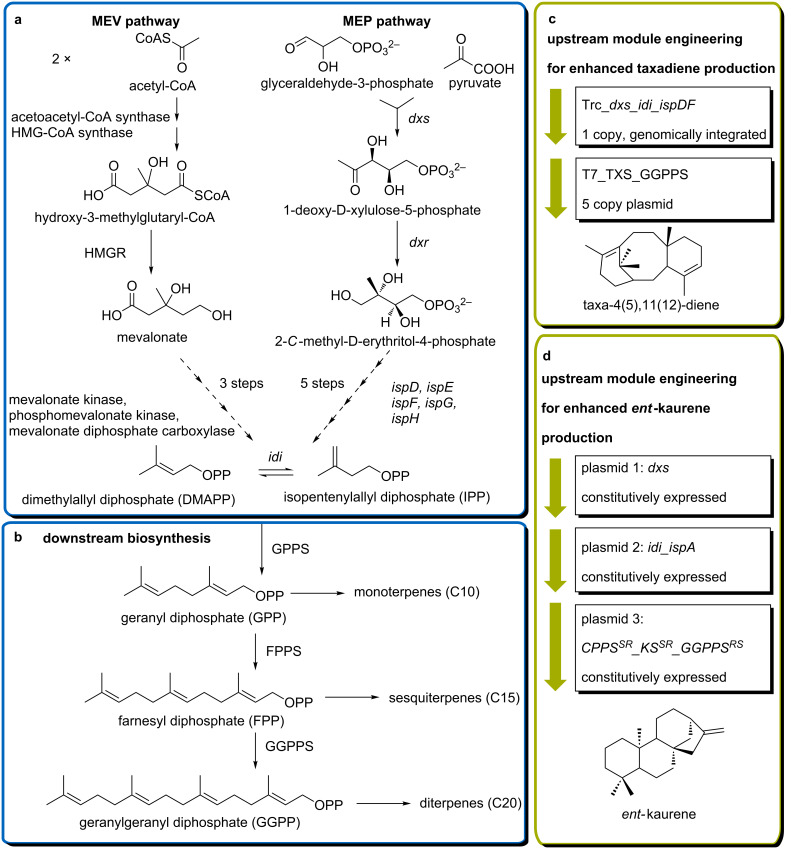
Isoprenoid biosynthetic pathways and examples for their engineering in heterologous production systems. a) Formation of central isoprenoid metabolites isopentenyl diphosphate (IPP) and dimethylallyl diphosphate (DMAPP) occurs via two distinct natural pathways. Designations MEV and MEP derive from significant intermediates: MEV = mevalonate-dependent and MEP= methylerythritol phosphate-dependent. b) Subsequent condensation of IPP and DMAPP by isoprenyl diphosphate synthases provides specific terpene synthases with their linear substrates. Terpenes are classified according to the carbon atom number in their basic scaffold, beginning with hemiterpenes (C5) and continuing in multiples of five. c) A possible strategy for MEP-pathway optimization for the improved production of the diterpene taxadiene reported by Ajikumar et al. [28]; targeted elements of the biosynthetic pathways and their expression manipulations are given. d) Selection of overexpression targets for the production of *ent*-kaurene reported by Kong et al. [29]; HMGR = hydroxymethylglutaryl(HMG)-CoA-reductase; *dxs* = 1-deoxy-D-xylulose-5-phosphate synthase; *dxr* = 1-deoxy-D-xylulose-5-phosphate reductoisomerase; *ispD* = 2-C-methyl-D-erythritol(ME)-4-phosphate cytidylyltransferase; *ispE* = 4-(cyt-5’-diphospho)-ME kinase; *ispF* = ME-2,4-cyclodiphosphate synthase; *ispG* = hydroxylmethylbutenyl(HMB)-4-diphosphate synthase; *ispH* = HMB-4-diphosphate reductase; *ispA* = farnesyl diphosphate synthase from *Escherichia coli*; *idi* = IPP isomerase; GPPS = geranyl diphosphate synthase; FPPS = farnesyl diphosphate synthase, GGPPS = geranylgeranyl diphosphate synthase; *GGPPS^RS^* = GGPPS from *Rhodobacter sphaeroides*; *KS^SR^* = *ent*-kaurene synthase from *Stevia rebaudiana*, *CPPS^SR^* = *ent*-copalyl diphosphate synthase from *Stevia rebaudiana*, Trc = Trc promoter; T7 = T7 promoter.

All terpenes derive from the ubiquitous central metabolites isopentenyl diphosphate (IPP) and dimethylallyl diphosphate (DMAPP) [24] (see Scheme 1). Interestingly, only two metabolic pathways (MEP and MEV) have been identified for the diverse biosynthesis of the structurally highly diverse family of isoprenoids. Both pathways use intermediate products of the central sugar metabolism as carbon sources [25]. In most eukaryotes (all mammals, yeast, fungi, archaea and plants (more precisely in the cytosol and mitochondria)) the isoprenoid precursors are synthesized via the mevalonate pathway (MVA) starting from acetyl-CoA [26]. Alternatively, in the majority of eubacteria, cyanobacteria, green algae and in the plastids of plants isoprenoid biosynthesis originates from glyceraldehyde-3-phosphate (G3P) and pyruvate [26–27]. Eponymous intermediate of this pathway is the product of the second enzymatic step where 1-deoxy-D-xylulose-5-phosphate (DXP) is reduced to 2-*C*-methyl-D-erythritol-4-phosphate (see Scheme 1).

Parallel occurrence of both pathways in higher plants is regulated through compartmentalization [30] with localization of diterpene biosynthesis in the plastids [31]. Metabolic engineering of plants to produce diterpenes remains challenging due to the required direction of biosynthetic enzymes into the specific organelles [32] and feedback inhibition of the 1-deoxy-D-xylulose-5-phosphate synthase (DXS) that can prevent accumulation of the desired lead structures [33].

### Isoprenyl diphosphate formation

Downstream of precursor formation condensation of IPP and DMAPP to longer-chain polyprenyls precedes subsequent metabolization to linear or mono- and polycyclic products, respectively, by the terpene synthases [24]. One exception to this standard sequence is presented by the hemiterpenes like isoprene which are directly derived from DMAPP [34].

In order to obtain mono-(C10), sesqui-(C15), di-(C20)terpenes and those harboring larger carbon skeletons, IPP and DMAPP are linked together by isoprenyl diphosphate synthases (IDSs) which are well-reviewed by Wang and Ohnuma [35]. Members of prenyltransferases are distinguished according to length and stereochemistry of their products [36–37].

*Z*-Isoprenyl diphosphate synthases are involved in the synthesis of very long-chain polyprenols like natural rubber [38] and the comparably short chains of dolichols [39]. The vast majority of terpenes, steroids and other isoprenoids like cholesterols and carotenoids are obtained from *E*-condensations [35].

Head-to-tail connection of single IPP and DMAPP by geranyl diphosphate synthase (GPPS) results in geranyl diphosphate, GPP, the universal precursor for all monoterpenes [40]. Subsequent *cis*-addition of further IPP-units to geranyl diphosphate by farnesyl diphosphate synthase (FPPS) and geranylgeranyl diphosphate synthase (GGPPS) yield in the respective precursors for sesquiterpenes (farnesyl diphosphate, FPP) and diterpenes (geranylgeranyl diphosphate, GGPP) [35].

### Terpene synthases

Interestingly, plant metabolism can convert the universal aliphatic diterpene precursor GGPP into thousands of different terpene structures with high structural complexity and elaborately functional decorations [41]. While the structural diversity of terpene products is obtained by precise modulation of cyclization and rearrangement steps performed by terpene cyclase enzymes [31], initial functional groups are introduced by hydroxylation of the carbon backbone with highly specific P450 monooxygenases [42–44].

At present, terpene synthases (TPS) are classified into three groups which mainly comprise α-helical structures that are designated as α-, β- and γ-domains [45]. Structural and catalytic diversity, especially of plant terpene synthases, originate in various combinations of these domains [46]. The three groups of terpene synthases are classed according to their intron/exon pattern [47] and their diverse reaction initiation mechanisms [48]. Genomic analyses of plant terpene synthases by Trapp and co-workers [47] revealed general organization of 12–14 introns for Class I terpene cyclases, 9 introns for Class II and 6 introns for Class III cyclases. Class III-type terpene synthases appear to be exclusively responsible for angiosperm secondary metabolites of mono-, sesqui- and diterpene structure and contain a highly conserved RR(x)8W-motif [47,49]. The terpene formation performed by Class I-type enzymes occurs via coordination of the isoprenyl diphosphate substrate by a three-ion cluster of divalent metal ions [48]. More specifically, Mg^2+^- or Mn^2+^-ions are bound by two conserved amino acid sequences, termed the DDXX(XX)D/E (“aspartate rich”) and NSE/DTE [(N,D)D(L,I,V)X(S,T)XXXE] motif, respectively [48]. The first committed step in synthesis of these Class I enzymes is the abstraction of the diphosphate group from the isoprenyl diphosphate substrate [50] at what the diphosphate group is postulated to remain inside the active site of the enzyme [51–52]. Class II terpene synthases harbor a distinct DXDD-motif [52] and the cyclization is generally initiated by protonation of the terminal carbon double bond of the substrate [53]. Since the diphosphate group is preserved during substrate activation by this type of synthases, products from Class II TPS can serve as substrates for Class I TPS which has been reported for example in the biosynthesis of labdane- and clerodane type diterpenes [41]. This close collaboration is performed either in one single bifunctional enzyme containing structure motifs of both types or sets of two different monofunctional synthases of both classes [54–55].

Engineering measures can directly target the primary structure of the terpene synthases or indirectly aim to alter or optimize the product spectrum by changing the tertiary or quaternary structure, respectively [23]. The following paragraphs should give an overview of selected current developments in the areas of mutational engineering, combinatorial enzyme design and microbial engineering.

### Mutational engineering of terpene synthases

**Scheme 2:**
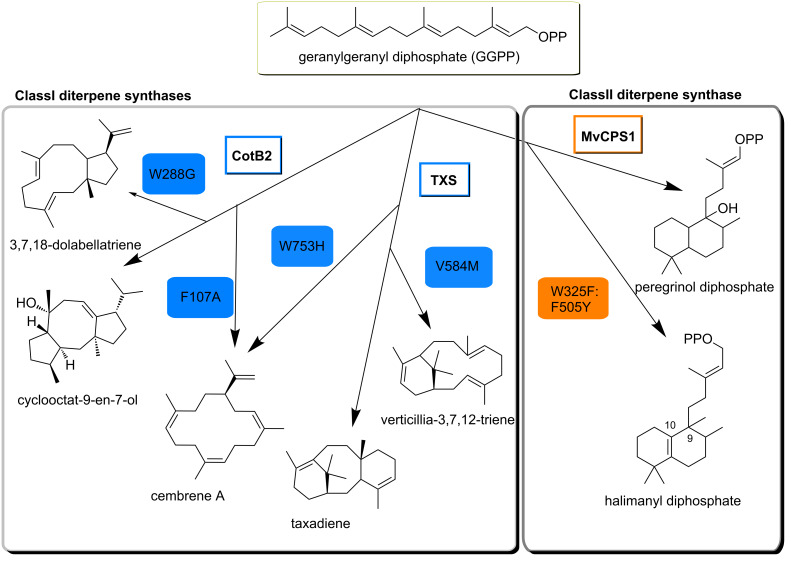
Mutational engineering of different classes of terpene synthases. Left side: The natural product of wild-type cyclooctat-9-en-7-ol-synthase (CotB2) is a tricyclic diterpene whereas mutations in positions 107 and 288 yield in monocyclic cembrene A and bicyclic 3,7,18-dolabellatriene [57]. Changing the main product specificity of taxadiene synthase from *Taxus brevifolia* (TXS) without significant loss in synthase activity was realized in bicyclic verticillia-3,7,12-triene production through mutation of valin584. Another mutation (W753H) resulted in 100% product specificity for cembrene A but TXS activity was reduced by half in comparison to the wild-type [51]. Right side: Methyl group shifts in Class II peregrinol diphosphate synthase from *Marrubium vulgare* (MvCPS1) were drastically rearranged by introduction of two mutations leading to a previously undescribed halimadane type diphosphate [58], a possible new precursor for valuable halimadane diterpenes with antimicrobial or anti-allergic potential.

Site-directed mutagenesis of diterpene cyclases is conventionally applied to elucidate structure–function relationships and mostly targets the active site of the enzyme in order to change the polarity or dimension of the substrate coordinating cavity. Recently reported targeted engineering [51] of the Class I taxadiene synthase from *Taxus brevifolia* (TXS) enabled new understanding of the mechanistic procedures that are carried out by this enzyme on the substrate GGPP. Quenching the carbocation cascade that naturally leads to the formation of tricyclic taxadiene [56] was achieved by exchanging a valin in position 584 with methionine. The resulting product was identified as a bicyclic diterpene of the verticillene type [51] (Scheme 2). A single residue switch in position 753 (W753H) presumably causes premature deprotonation of a cembrene-15-yl cation intermediate in the cyclization mechanism of TXS leading to the monocyclic cembrene A [51] (Scheme 2).

Hence reprogramming the catalytic cascade of diterpene synthases and subsequent functional expression of enzyme variants in a microbial host can not only provide insights into cyclization mechanism but also lead to novel products or changes in the product spectra. This has also been demonstrated for the bacterial diterpene cyclooctat-9-en-7-ol synthase (CotB2) [57], also a putative Class I TPS. Mutation of tryptophan 288 to glycine in CotB2 resulted in the stereoselective synthesis of (1*R*,3*E*,7*E*,11*S*,12*S*)-3,7,18-dolabellatriene, a bicyclic diterpene (Scheme 2). Dollabellanes derive mostly from marine organisms and display bioactivities such as antiviral and cytotoxic effects [59]. Dolabellatriene from a reprogrammed CotB2 contribute with antimicrobial activity against multidrug resistant *Staphylococcus aureus* to this family of natural products [57]. Other mutations on this enzyme generated one cembrane-type monocycle (F107A) (Scheme 2) and two non-natural fusicoccane-type diterpenes (F107Y and F149L) [57]. The latter are putative intermediates in novel routes to phytotoxic fusicoccin A [60] and its derivative with presumably anticancer potential [61].

Exchange of two amino acid residues in the active site of the Class II peregrinol diphosphate synthase from the horehound *Marrubium vulgare* (MvCPS1) [58] resulted in an altered neutralization mechanism of a labda-13-en-8-yl diphosphate carbocation intermediate and the formation of halima-5(10),13-dienyl diphosphate (Scheme 2). In wild-type Class II diterpene synthases, the labda-13-en-8-yl diphosphate carbocation undergoes either single deprotonation or a cascade of hydride and methyl group shifts prior to deprotonation with occasional hydration at the C8 position of the carbocation intermediate which yields hydroxylated diphosphate products [62]. MvCPS1, however, catalyzes a C9–C8 hydride shift preceding hydration resulting in the labdane-type diterpene precursor for the antidiabetic marrubiin [63]. Double mutations of MvCPS1, W323L:F505Y, and W323F:F505Y completely changed the product specificity towards a novel, so far uncharacterized halimadane type diterpene [58] (Scheme 2).

### Combinatorial biosynthesis – enzyme design for manufactured terpenes

Conventional identification of new enzyme activities involved in diterpene biosynthetic routes entail time-consuming genome-mining and high-throughput screening technologies [64–65]. Additionally, the number of currently available, even partly annotated plant genomes and crystal structures of diterpene synthases is still limited. Yet, in order to establish heterologous production systems for known diterpenes or to obtain new compounds, deep understanding and accessibility to structural information of this enzyme class can be crucial.

In the last few years, modular approaches encompassing metabolomics and transcriptomics-based methods opened up new avenues for the rapid identification of (di)terpenes.

Andersen-Ranberg and co-workers reported recently on the creation of a synthetic collection of monofunctional Class I/Class II diterpene synthase combinations, which lead to high stereoselective syntheses of an impressive number of previously unknown or unamenable diterpenes with labdane- and clerodane-type structures [66]. Additional findings were provided by Jia and co-workers [67], who demonstrated high substrate promiscuity of a plant and a fungal Class I diterpene synthase. This study involved general substrates of diterpene cyclases like GGPP and its *cis*-isomer nerylneryl diphosphate (NNPP) [68] but also new combinations with 12 known and available products of plant Class II diterpene synthases. Consequently, they obtained 13 previously undescribed diterpenes of the labdane family in addition to previously described diterpenes like manool [69], sclareol [69] and *cis*-abienol [64].

A biosynthesis study of salvinorin A (a psychotrophic agent with potential application as neuropsychiatric drug and for addiction treatment) in *Salvia divinorum* [70] resulted in the identification of five new Class I and Class II diterpene synthases. Moreover, this study performed in vivo substrate promiscuity tests following a combinatorial approach [41,66]. The resulting products entailed pimarane- and abietane-type diterpenes as well as the *trans*-clerodane type diterpene kolavenol, a putative intermediate in the salvinorin A biosynthesis.

Other bifunctional diterpene synthases do not comprise combinations of Class I/Class II domains but contain both a prenyltransferase domain and a terpene synthase moiety. This combination of catalytic modules allows the direct formation of the isoprenyl diphosphate substrate for the terpene synthase in a single biocatalyst. An unusual example of these bifunctional enzymes was published by Chen and coworkers [60], who managed to crystalize catalytic domains of PaFS, a diterpene synthase from *Phomopsis amygdali*. The formation of GGPP is located in a C-terminal α-domain with very low sequence identity to the N-terminal Class I terpene synthase domain indicating different catalytical properties. The natural product of PaFS is fusicocca-2,10(14)-diene, an intermediate in the biosynthesis of the phytotoxin fusicoccin A by *P. amygdali*. Interestingly, a recent work by Qin and co-workers [71] even revealed the conversion of a fungal diterpene synthase into a sesterterpene synthase by interchanging the prenyltransferase domain.

Combining these structural insights and newly created biosynthetic routes with functional expression in bacterial production hosts, industrial scale synthesis of fragrance compound (+)-sclareol, (13*R*)-(+)-manoyl oxide (precursor for pharmaceutic forskolin) or miltiradiene (precursor for antioxidants and tanshinones) may be within reach [66]. Additionally, genetic engineering of diterpene synthases enhances the knowledge of structure–function relationships alongside with increasing supply of novel potentially bioactive diterpenes.

Reprogramming the catalytic activities of (plant) diterpene synthases may also be an alternative to extensive genome-mining and screening strategies since this technique can potentially close or circumvent knowledge gaps in biosynthetic pathways to bioactive products which were previously inaccessible. A good example is the mutagenesis of the bacterial diterpene synthase CotB2 that resulted in dolabellatriene-type scaffolds, which were by then mostly found in marine organisms [59]. To that end, these new routes can provide substantial and environmentally friendly alternatives for sourcing natural diterpenes from rare resources like corals [72].

### Microbial engineering

The genetically readily accessible engineering hosts *Escherichia coli* and *Saccharomyces cerevisiae* are suitable for heterologous terpene production. Established culture conditions are completed by the availability of metabolic databases and computational tools [73] that enable model-based optimization such as flux-balance analyses [74]. Taking advantage of natural presence and manipulability of distinct isoprenoid pathways in both organisms (MEP in *E. coli* and MEV in *S. cerevisiae*), heterologous production of several isoprenoid natural products has been accomplished with industrially relevant production titers [75–77]. However, at present there is no clear preference for one microbial production host as each engineering endeavor requires a de novo benchmarking for a specific terpenoid product, indicating that even minimal introduction of heterologous genes for terpene production lead to unpredictable metabolic feedback reactions that currently can only be counteracted by empirical approaches. Monoterpenes, for example, can have toxic effects on microorganisms, though *E. coli* seems to be more tolerant towards products like α-pinene or limonene [78].

At present, 27.4 g/L of amorphadiene is the highest published titer for any reported terpenoid produced in *E. coli.* This result is of particular industrial relevance as amorphadiene constitutes the sesquiterpenoid scaffold of the antimalarial drug artemisinin [79]. In comparison, production of amorphadiene in *S. cerevisiae* did yield in excess titer of 40 g/L [80].

The opposite result to amorphadiene was observed for the model diterpene taxadiene where heterologous production in *S. cerevisiae* resulted in 8.7 mg/L, while yields of 1 g/L taxadiene could be obtained in *E. coli* [28,81]. Different approaches of terpene product increase involved targeting specific elements of the MEP pathway [28] or introducing heterologous MEV pathway from yeast [82–83]. A high level of taxadiene production in *E. coli* was achieved by Ajikumar and co-workers through overexpression of bottleneck enzymes of the endogenous MEP pathway (*dxs*, *idi*, *ispD*, *ispF*, Scheme 1) together with GGPP-synthase (GGPPS) and taxadiene-synthase (TXS) from *Taxus brevifolia* [28]. Elevation of product titers of the important diterpene intermediate *ent*-kaurene (precursor for the gibberellin biosynthesis [84]) was reported from Kong et al. [29]. Their strategy involved overexpression of the MEP-Elements *dxs, idi* and *ispA* (see Scheme 1) in an engineered *E.coli* strain co-expressing recombinant *ent*-copalyl diphosphate synthase (CPPS) and *ent*-kaurene synthase (KS) from *Stevia rebaudiana* as well as a GGPPS from *Rhodobacter sphaeroides*.

With an increasing number of integrated recombinant enzymes balanced (over)expression gains importance in order to sustain optimal carbon flux in the production host from cultivation medium feed to the desired product. In this respect, determining the optimal strength of the ribosomal binding site (RBS) may be as crucial as the correct arrangement of the genetic elements on designed operons [85–87]. To this end, the lycopene reporter system represents a valuable tool in determining balanced expression of terpene centered heterologous pathways in *E. coli* [88–89]. Furthermore, a significant obstacle in large scale bacterial diterpene production is the functional expression of engineered terpene synthases in the heterologous host. Similarly, the downstream functionalization of the hydrocarbon scaffold, which is a prerequisite for biological activity [90], remains challenging in any recombinant host. The vast majority of modifications accomplished in the downstream biosynthesis of diterpenes comprise introduction of oxygen moieties by cytochrome-P450 enzymes, which are commonly not sourced from bacterial systems. In fact the functional reconstitution of eukaryotic terpene synthases or oxidoreductases requires significant enzyme modifications. Specifically, codon optimization and the truncation of distinct domains which are responsible for, e.g., membrane localization can improve the enzyme activity. To date, identifying the necessary sequence segment for soluble expression in the bacterial host alongside with finding the optimal redox partner for P450 enzymes is still a matter of empirical work [44,91–92]. Furthermore, integration of every additional enzyme to the production system will eventually result in a significant decrease in the final yield, which makes very complex biosyntheses involving multiple oxygenation steps challenging [93]. Even highly optimized systems for the production of just the first hydroxylated intermediate in taxol biosynthesis, 5-α-hydroxytaxadiene [91], show a product loss of over 40% in comparison to previously reported titers for the undecorated taxadiene macrocycle [28]. Engineering one host alone can therefore sometimes be insufficient since there are specific elements of biosynthetic pathways that may have different production capacities in one organism or another. Zhou et al. [94] reported stable co-culture fermentation of specifically engineered *E. coli* and *S. cerevisiae* strains for the production of different sesqui- and diterpenes. Although, the final yield for the taxane product was in very low mg scale, first microbial production of deoxygenated monoacetylated taxadiene could be realized.

Optimization of the up-scaled fermentative process generally involves selection of the carbon source, media composition and in situ or post-fermentational product removal. Some terpene products have cytotoxic effects against the production host and separation from the cells is recommended already during fermentation. Ajikumar et al. also reported in-process-accumulation of the inhibitory metabolite indole [28]. A suitable method for most fermentations is an overlay with apolar alkanes such as dodecane [28,79,95] although subsequent product extraction from this phase may be challenging and oxygenation capacity is reduced. Engineering efflux transporters to enhance extracellular product secretion can be a viable support for this apolar-phase-capture [96]. However, these methods are no longer applicable as soon as further engineering steps involve polarization of the product backbone.

**Figure 1:**
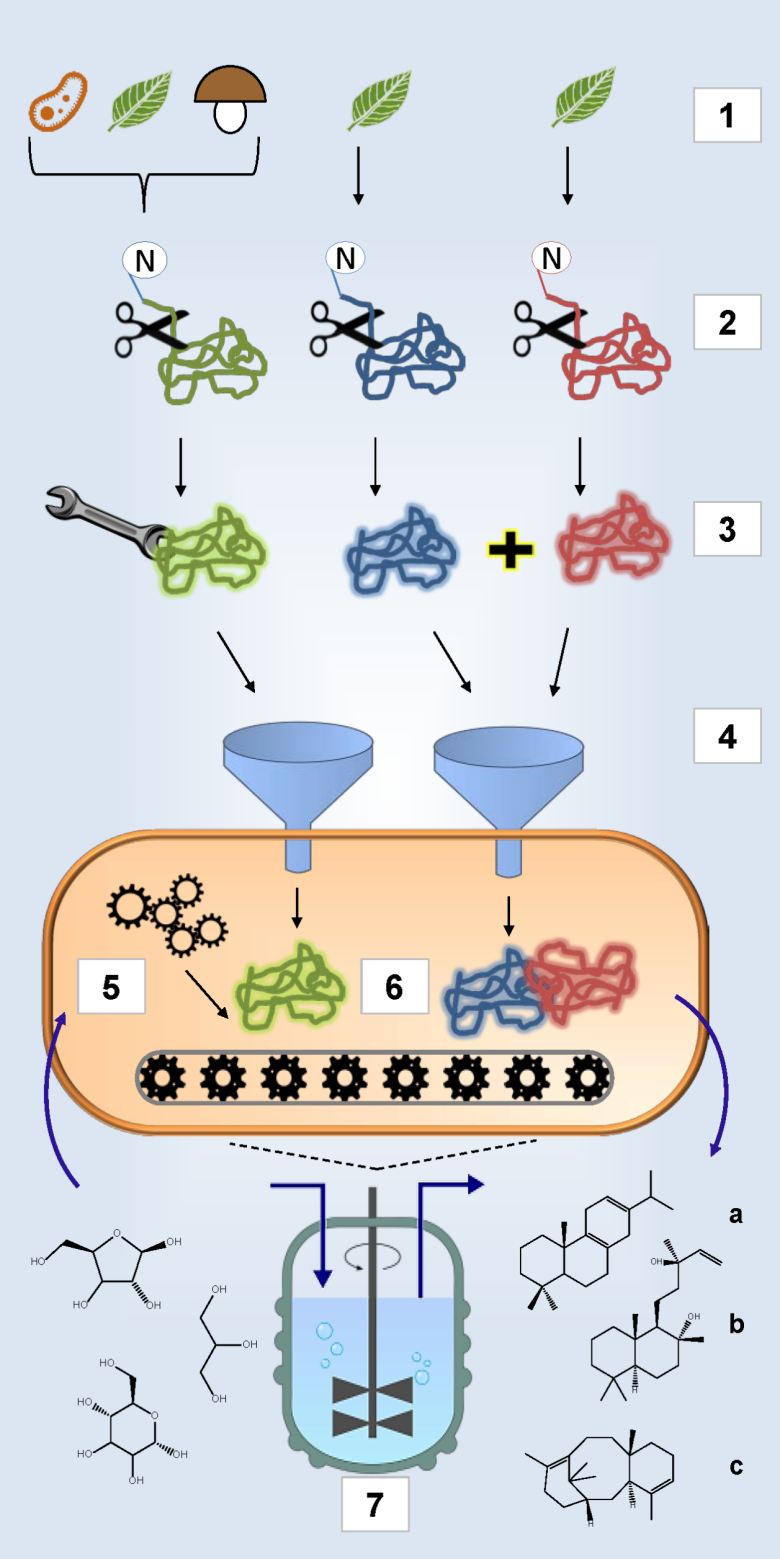
Implementation of a microbial cell factory. 1: Selection of enzymes from different species. P450 and related reductase enzymes (indicated with blue and red knots respectively) derive almost exclusively from plants; terpene synthases and other enzymes that are involved in precursor formation (indicated with green knot) can be obtained from various organisms (indicated through symbolic bacteria, leaf (representing plants in general) and fungi). 2: Eukaryotic enzymes have to be engineered for functional and soluble expression in prokaryotic hosts like *E. coli*, Removal of the N-terminal and thereby the cell-wall-localization domain (indicated through scissors) is a standard procedure in engineering plant enzymes; 3: Further engineering steps are not mandatory but often entail site-directed mutagenesis (indicated through wrench) of TPS (green) for product modulation or introduction of a linker-coding sequence for co-expression of P450 monooxygenase and reductase (blue and red); 4: Heterologous expression in *E. coli* (depicted in orange). Construction of synthetic operons and screening for highest yield of plasmid systems generally precedes genomic integration; 5: Isoprenoid precursor supply: precursor flux has to be balanced carefully to avoid metabolic overload and accumulation of unwanted byproducts; 6: Downstream terpenoid biosynthesis using heterologous enzymes; 7: Upscaling of terpene production in fermentation systems using different carbon sources (left) for optimally engineered *E. coli* strains is a potential future source for valuable diterpenes like miltiradiene (a), sclareol (b) or taxadiene (c).

A summary of the various areas that have to be covered for successful establishment of heterologous terpene production in a bacterial host is given in Figure 1.

## Conclusion

Over the last years, countless and in some cases ground-breaking studies about terpenes and heterologous terpene biosynthesis have been published, and it still seems like just the tip of the iceberg. Potentially, modular biosynthesis that has resources to fast expanding databases will widen the amenable targets for large scale production to unforeseen extend. As a prerequisite, strain optimization of heterologous hosts has to be developing with equal progress although continuous reporting about engineering the native pathway for isoprenoid precursor formation in *E. coli*, MEP, proves its complexity. Computational approaches that involve flux-balance analyses can redirect empirical screening for optimized systems towards guided engineering to overcome metabolic bottlenecks and identify feedback inhibition loops. Heterologous production of several diterpenes could already be realized in stable systems with moderate yields, validating the established approaches of enzyme engineering for terpene synthases. Yet this success could not be transferred in full extend to heterologous expression of P450 enzymes. Solubility together with substrate and product specificity remains important targets for further engineering.
